# Genome Sequence of “*Candidatus* Dehalogenimonas etheniformans” Strain GP, a Vinyl Chloride-Respiring Anaerobe

**DOI:** 10.1128/MRA.01212-20

**Published:** 2020-12-10

**Authors:** Yi Yang, Jun Yan, Xiuying Li, Yan Lv, Yiru Cui, Fadime Kara-Murdoch, Gao Chen, Frank E. Löffler

**Affiliations:** a Key Laboratory of Pollution Ecology and Environmental Engineering, Institute of Applied Ecology, Chinese Academy of Sciences, Shenyang, Liaoning, China; b University of Chinese Academy of Sciences, Beijing, China; c Center for Environmental Biotechnology, University of Tennessee, Knoxville, Tennessee, USA; d Department of Microbiology, University of Tennessee, Knoxville, Tennessee, USA; e Department of Civil and Environmental Engineering, University of Tennessee, Knoxville, Tennessee, USA; f Department of Biosystems Engineering and Soil Science, University of Tennessee, Knoxville, Tennessee, USA; g Biosciences Division, Oak Ridge National Laboratory, Oak Ridge, Tennessee, USA; h Joint Institute for Biological Sciences (JIBS), Oak Ridge National Laboratory, Oak Ridge, Tennessee, USA; University of Maryland School of Medicine

## Abstract

“*Candidatus* Dehalogenimonas etheniformans” strain GP couples growth with the reductive dechlorination of vinyl chloride and several polychlorinated ethenes. The genome sequence comprises a circular 2.07-Mb chromosome with a G+C content of 51.9% and harbors 50 putative reductive dehalogenase genes.

## ANNOUNCEMENT

Members within the genus *Dehalogenimonas* are obligate organohalide-respiring bacteria implicated in the turnover of naturally occurring and anthropogenic chlorinated compounds in anoxic environments (1, 2). An enrichment culture capable of dechlorinating vinyl chloride (VC) and several polychlorinated ethenes to ethene was established with grape pomace compost as the source material (3). Proteogenomics analysis implicated a novel *Dehalogenimonas* bacterium, strain GP, with 95.3 to 99.5% 16S rRNA gene sequence similarity to available *Dehalogenimonas* isolates (4–6). A dilution-to-extinction procedure in defined mineral salt medium containing 1 g liter^−1^ ampicillin, 0.1 g liter^−1^ vancomycin, acetate as the carbon source, hydrogen as the electron donor, and 1,1-dichloroethene as the electron acceptor was used to achieve isolation of strain GP (7).

Genomic DNA from strain GP biomass was extracted using the cetyltrimethylammonium bromide method (8). For Nanopore sequencing, large fragments of genomic DNA were obtained using the Blue Pippin automatic nucleic acid fragment recovery system (Sage Science, MA), and barcodes were added using the Nanopore EXP-NBD104 kit. Automated capillary electrophoresis was used to determine the fragment sizes before a library with an average insert size of 10 kb was constructed using the Nanopore SQK-LSK109 ligation kit, and sequencing was performed with a Nanopore PromethION system (9). The fast5 file was transformed using Nanopore’s Guppy software, and quality control was performed using the NanoPlot plotting tool (10) with a threshold value of Q > 7. The 87,547 Nanopore reads represent a total length of 560,844,341 bp and have a median read length of 6,406 bp and an *N*_50_ read length of 10,114 bp. For Illumina sequencing, DNA was sonicated to generate fragment lengths of <350 bp, followed by end polishing and T-A ligation before library preparation using a NEBNext Ultra DNA library prep kit (New England Biolabs, Ipswich, MA). Paired-end sequencing (2 × 150 bp) was performed using a NovaSeq PE150 flow cell (Illumina, San Diego, CA). The sequence reads were trimmed and filtered (https://github.com/lh3/readfq), resulting in 4,073,240 high-quality reads. Assembly using NanoPore raw long reads (coverage, 271×) and Illumina short reads (coverage, 591×) was performed using Unicycler version 0.4.7 (11) and polished using Pilon version 1.20.1 (12). Unicycler version 0.4.7 was used to identify overlapping contig ends, indicating chromosomal circularization. Gene prediction and functional annotation were performed using the NCBI Prokaryotic Genome Annotation Pipeline (13).

The genome comprises one circular 2,068,322-bp chromosome with a G+C content of 51.9%. The genome contains 2,029 coding sequences, 47 tRNA genes, and single copies of the 5S, 16S, and 23S rRNA genes. The GP genome harbors 50 nonidentical reductive dehalogenase (*rdh*) subunit A genes, including *cerA* (locus tag number HX448_10020), which encodes a VC *rdh* (3). Four of the fifty putative *rdhA* genes are adjacent to a downstream *rdhB* gene encoding a predicted membrane anchor. The GP genome harbors two formate dehydrogenase genes, locus tag numbers HX448_03659 and HX448_07080, the former encoding a protein with a selenocysteine at position 193 and 90.65% amino acid identity to formate dehydrogenase (Dform_00419) of *D. formicexedens* strain NSZ-14^T^ (6). Average nucleotide identity (ANI) analysis using JSpeciesWS version 3.5.1 (14) determined that strain GP shares 78.39%, 71.46%, 69.51%, and 68.61% ANI with *D. formicexedens* strain NSZ-14^T^ (GenBank accession number CP018258.1), *D. alkenigignens* strain IP3-3^T^ (shotgun sequencing project accession number LFDV00000000.1), *Dehalogenimonas* sp. strain WBC-2 (GenBank accession number CP011392.1), and *D. lykanthroporepellens* strain BL-DC-9^T^ (GenBank accession number CP002084.1), respectively. The new data expand the *Dehalogenimonas* pangenome and *rdh* sequence diversity.

### Data availability.

The genome has been deposited at the DNA Data Bank of Japan, the European Nucleotide Archive, and GenBank (accession number CP058566.1). The BioSample and BioProject accession numbers are SAMN15398252 and PRJNA258024, respectively. The raw reads were deposited in the Sequence Read Archive under accession numbers SRR12774736 (Nanopore) and SRR12774735 (Illumina).
